# A Roadmap to Strengthen Caregiver Services and Supports in California

**DOI:** 10.1093/geroni/igaf122.999

**Published:** 2025-12-31

**Authors:** Rachael Fulp-Cooke, Janice Bell, Shikha Bhurtel, Quynh Vo, Pauline Martinez, Lorena Garcia, Kathleen Kelly, Heather Young

**Affiliations:** Betty Irene Moore School Of Nursing at UC Davis, Sacramento, California, United States; Betty Irene Moore School Of Nursing at UC Davis, Sacramento, California, United States; University of California, Davis, Davis, California, United States; University of California, Davis, Davis, California, United States; Betty Irene Moore School of Nursing, University of California, Davis, Davis, California, United States; Betty Irene Moore School Of Nursing at UC Davis, Sacramento, California, United States; Family Caregiving Alliance, San Francisco, California, United States; Betty Irene Moore School Of Nursing at UC Davis, Sacramento, California, United States

## Abstract

Family caregivers provide essential support for older adults and people with disabilities across California. However, many caregivers face barriers to accessing programs and services that support their own health and well-being. Demographic shifts and increasing care demands are reshaping California’s caregiving landscape. To address these challenges, the California Department of Aging (CDA) partnered with the Family Caregiving Institute (FCI) at UC Davis to develop the California Caregiver Equity Roadmap (“Roadmap”). This initiative aims to improve publicly funded caregiver services and supports in California and advances the fourth goal of the Master Plan for Aging: Caregiving That Works. Community engagement was central to the Roadmap’s development. Input was regularly collected from a wide range of constituent groups including a statewide advisory group, a steering committee, and leadership from Caregiver Resource Centers and Area Agencies on Aging. These constituent groups were comprised of family caregivers and individuals from caregiving service organizations. Strategic goals and actions were developed, tested, refined, and revised through an iterative process including regular meetings and two surveys distributed to constituents statewide. The final Roadmap outlines five strategic goals and 24 supporting actions to strengthen California’s caregiving infrastructure, focusing on five key areas: 1) awareness, 2) access, 3) supporting health and social service providers, 4) using evidence to strengthen services and supports, and 5) using data to inform service improvement. Research implications include evaluating the Roadmap’s impact on caregiving services and supports. Findings could serve as a model for other states aiming to strengthen family caregiver support.

